# Gene expression studies in Depression development and treatment: an overview of the underlying molecular mechanisms and biological processes to identify biomarkers

**DOI:** 10.1038/s41398-021-01469-6

**Published:** 2021-06-08

**Authors:** Nicole Mariani, Nadia Cattane, Carmine Pariante, Annamaria Cattaneo

**Affiliations:** 1 Stress, Psychiatry and Immunology Laboratory, Department of Psychological Medicine, Institute of Psychiatry, Psychology & Neuroscience, King’s College London, London, UK; 2 IRCCS Istituto Centro San Giovanni di Dio Fatebenefratelli, Biological Psychiatry Laboratory, Brescia, Italy; 3 Department of Pharmacological and Biomolecular Sciences, University of Milan, Milan, Italy

**Keywords:** Psychology, Biomarkers, Depression

## Abstract

A combination of different risk factors, such as genetic, environmental and psychological factors, together with immune system, stress response, brain neuroplasticity and the regulation of neurotransmitters, is thought to lead to the development of major depressive disorder (MDD). A growing number of studies have tried to investigate the underlying mechanisms of MDD by analysing the expression levels of genes involved in such biological processes. These studies have shown that MDD is not just a brain disorder, but also a body disorder, and this is mainly due to the interplay between the periphery and the Central Nervous System (CNS). To this purpose, most of the studies conducted so far have mainly dedicated to the analysis of the gene expression levels using postmortem brain tissue as well as peripheral blood samples of MDD patients. In this paper, we reviewed the current literature on candidate gene expression alterations and the few existing transcriptomics studies in MDD focusing on inflammation, neuroplasticity, neurotransmitters and stress-related genes. Moreover, we focused our attention on studies, which have investigated mRNA levels as biomarkers to predict therapy outcomes. This is important as many patients do not respond to antidepressant medication or could experience adverse side effects, leading to the interruption of treatment. Unfortunately, the right choice of antidepressant for each individual still remains largely a matter of taking an educated guess.

## Introduction

Major depressive disorder (MDD) is a complex psychiatric disorder characterized by low mood, anhedonia, feelings of guilt or low self-worth, disturbed sleep or appetite, low energy and suicidal ideation^1,2^. It is one of the main causes of disability worldwide and is a major contributor to the overall global burden of disease^3^.

The combination of genetic, environmental and psychological factors is believed to be the cause of MDD. In fact, for instance, environmental factors, such as stressful and traumatic events, can affect not only biological systems restricted to the brain, but also pathophysiological pathways within the entire body^4,5^. Well-established evidence suggests deregulation in the inflammatory response, in the hypothalamus-pituitary-adrenal (HPA) axis and in several neuronal systems in the pathogenesis of MDD^6,7^. As such, acute and chronic stress have been proposed to trigger the dysregulation of these systems and to lead to the development of MDD^8^. Hence, biological systems such as immune system, stress response, brain neuroplasticity and the regulation of neurotransmitters seem to be the ones more involved in MDD.

To date, different approaches have been used to understand the molecular mechanisms underlying MDD. Among the others, gene expression is being used in a large number of studies to analyse the expression of dozens of genes in MDD.

To this purpose, most of the studies conducted so far have mainly investigated the gene expression levels using postmortem brain tissue^9^ as well as peripheral blood samples of MDD patients. While the use of brain tissue is limited and has several limitations due to the influence of agonal and postmortem factors on gene expression levels^10^, the use of peripheral blood samples seems to have multiple advantages. Indeed, peripheral blood samples allow to collect large sample sizes, to obtain a fast RNA stabilization, as well as the isolation of specific cell subtypes, such as peripheral blood mononuclear cells (PBMCs) or leukocytes and to monitor the patients’ well-being.

The association between the brain and the periphery has been demonstrated several years ago by Sullivan and colleagues^11^, who have shown genes shared among whole blood and 16 brain tissues, where 60% of transcripts were expressed in the whole blood and in at least one tissue of the central nervous system (CNS). In detail, both whole blood and brain tissues have similar expression of genes relevant to MDD such as genes encoding for neurotransmitter receptors and transporters, growth factors, hormones and cytokines. In addition to these data, transcriptional profiling in peripheral blood has allowed the discovery of possible biomarkers for patients with psychiatric and neurological disorders including patients affected by MDD^12–14^.

Based on this, we reviewed the current literature on candidate gene expression in MDD, mainly focusing on genes related to inflammation, neuroplasticity, neurotransmitters, stress response and treatment outcomes. We have also included a few existing transcriptomics studies, which identified changes in gene expression levels by using a hypothesis-free approach.

Blood gene expression alterations in MDD have been already reviewed in 2013^15^ by our group. Although in the paper by Hepgul et al. we focused on inflammation, GR functionality and neuroplasticity, we did not report gene expression studies in relation to treatment outcome. Since in these last years a large body of studies has investigated gene expression alterations in association with MDD from 2013 to date, also including treatment outcomes, we have seen the need for a more up-to-date review.

## Inflammation-related genes

In recent years, several studies have suggested an increased inflammatory response in MDD, indicated by altered levels of pro- and anti-inflammatory cytokines^16,17^. Furthermore, other studies have linked several autoimmune diseases, such as multiple sclerosis, rheumatoid arthritis, multiple sclerosis and inflammatory bowel diseases, with MDD, suggesting a very strong relationship between inflammation and MDD^18,19^. However, although it is well known that depression can influence immune responses and vice versa, the underlying molecular mechanisms are still unclear.

Among all the molecules involved in the immune response, cytokines, known as chemical messengers between immune cells, represent the most important key players in mediating depressive symptoms. They include various groups of molecules produced, upon stimulation by pathogens or dysfunctional cells, by immune cells of the periphery as well as cells of the central nervous system such as microglia, astrocytes, oligodendrocytes. Moreover, also neurons can release cytokines and chemokines as well as respond to them through cytokine and chemokine receptors^20^.

For this reason, also taking into account that cytokines can cross the blood–brain barrier^21^, they may represent a potentially useful biomarker resource relating to mood disorders.

Several components of the immune system, including the Toll-like receptors (TLRs), their intracellular signaling molecules and their related pro-inflammatory transcription factors such as nuclear factor kappa-light-chain-enhancer of activated B cells (NF-kB) and interferon regulatory transcription factor 3 (IRF3) play crucial roles in the production of pro-inflammatory cytokines, including Interleukin (IL)-1b and IL-18^22^.

To investigate the role of inflammation in MDD, several studies available so far have measured the mRNA levels of genes involved in inflammation in the peripheral blood and postmortem brain tissues of patients with MDD (see Table 1). For example, the study conducted by Rizavi et al. in 2016^23^ indicated an increased expression of pro-inflammatory cytokines and their receptors in the lymphocytes of depressed patients as compared to control subjects, proposing an abnormal expression not only of genes encoding for pro-inflammatory cytokines, but also of genes encoding for their membrane-bound receptors in MDD. Moreover, Momeni et al.^24^ showed higher mRNA levels of an adaptor protein (ASC), correlated with absent in melanoma 2 (AIM2) gene, in peripheral blood of depressed patients. AIM2 is a component of inflammasomes, which can trigger caspase-1 via ASC following a pathogen-associated molecular pattern (PAMP) or danger-associated molecular pattern (DAMP) recognition. Therefore, the activation of caspase-1 can trigger the induction of IL-1 and IL-18, two important pro-inflammatory cytokines. Similarly, the Genome-Based Therapeutic Drugs for Depression (GENDEP) project showed that the mRNA expression of inflammation-related genes, such as IL-1b, macrophage inhibiting factor (MIF) and tumor necrosis factor (TNF) are higher in non-responders depressed patients before treatment^25^.

**Table 1:** Studies examining alterations in the expression of inflammation-related genes.

Citation	Sample	Methods	Gene	Main findings	Tissue
Cattaneo et al. 2020^38^	130 MDD patients: 36 treatment-responsive 36 drug-free 58 treatment-resistant 40 healthy controls	RT-qPCR	IL-1b, IL-6, MIF, TNF-a, P2RX7, CCL2, CXCL12, AQP4, ISG15, STAT1, USP18	All genes, except AQP4, ISG15 and USP18, were differentially regulated. Treatment-resistant and drug-free depressed patients had evidence of increased inflammasome activation (higher pro-inflammatory cytokines/chemokines and P2RX7 mRNAs expression) compared with treatment-resistant and controls.	Peripheral blood
Spindola et al. 2017^26^	20 children and adolescents with MDD, 49 participants without MDD diagnosis but with high levels of depressive symptoms (DS), 61 healthy controls	RT-qPCR	TNF, TNFR1, IL-1b	Decreased mRNA levels of NR3C1, TNF, TNFR1 and IL-1b in MDD group compared with controls and DS group.	Peripheral blood
Iacob et al. 2013^41^	23 females with medication refractory DD: 13 MDD patients 10 with BPD19 healthy controls	RT-qPCR	IL-10, IL-6, TNF	Increased expression of IL-10, IL-6 in DD patients. BPD patients showed decreased TNF expression compared with controls. Depression severity was related to increased IL-10 expression when compared with controls.	Peripheral blood leukocytes
Talarowska et al. 2014^42^	131 rDD patients 105 healthy controls	RT-qPCR Spectrophotometer GeneQuest	MnSOD, SOD2	MnSOD gene expression at mRNA and protein level was significantly lower in rDD patients than in the HC group.	Blood serum
Yang et al. 2017^43^	8 MMD patients 8 SSD patients 8 healthy controls	RT-qPCR	CD84	Expression of CD84 was significantly increased in MMD and SSD compared with control.	Peripheral blood
Amidfar et al. 2017^37^	25 medication naïve-patients with MDD 25 medication-free MDD patients 25 healthy controls	RT-qPCR	5-HT2A, 5-HT3A	5-HTR2A mRNA expression was significantly higher in PBMCs of all MDD patients when compared with healthy controls. No significant difference in the relative levels of 5-HTR3A mRNA expression in PBMCs of all MDD patients when compared with healthy controls.	PBMC
Bobińsa et al. 2016^44^	139 rDD patients 95 healthy controls	RT-qPCR	MMP-2, MMP-9	Decreased expression of MMP-2 and MMP-9 genes on both mRNA and protein levels in depression when compared with controls.	Peripheral blood
Cattaneo et al. 2013^25^	811 MDD patients: 51 responders 23 non-responders 34 healthy controls	RT-qPCR	IL-1a, IL-1b, IL-4, IL-6, IL-7, IL-8, IL-10, TNF-a, MIF	Depressed patients, as compared with controls had higher mRNA levels of IL-1b, IL-6, MIF and TNF-a, and lower levels of IL-4.	Peripheral blood
Hajebrahimi et al. 2014^22^	38 MDD students 43 healthy controls	RT-qPCR	TRIF, MYD88	mRNA expression levels of TRIF and MYD88 were increased on MDD when compared with control.	PBMC
Hung et al. 2014^45^	30 MDD patients 29 healthy controls	RT-qPCR	TLRs	Higher TLR3, 4, 5 and 7 mRNA expression levels in patients with MDD compared with controls, whereas no significant differences for TLR2, 8 or 9 were observed. Lower expressions of TLR1 and TLR6 in patients with MDD when compared with healthy controls.	Peripheral blood
Momeni et al. 2016^24^	38 MDD students 43 healthy controls	RT-qPCR	AIM2, ASC	mRNA levels of AIM2 were similar in both groups. ASC levels were significantly increased in MDD patients when compared with controls.	PBMC
Lukic et al. 2014^35^	30 MDD patients 35 healthy control	Western Blot	Nrf2, NF-κB, MnSOD, CuZnSOD, CAT	Upregulation of redox-sensitive transcriptional factors (Nrf2 and NF-κB) and AOEs (MnSOD, CuZnSOD and CAT) in MDD patients when compared with controls.	PBMC
Pantazatos et al. 2016^46^	21 MDD- suicides patients 9 MDD patients 29 healthy controls	RT-qPCR	IL-8, CCL4	IL-8 and chemokine ligand 4 (CCL4) analyses revealed lower expression in depressed and/or suicide patients than in healthy controls.	Postmortem brain tissues
Powell et al. 2014^47^	40 BPD patients 45 MDD patients 40 healthy controls	RT-qPCR	IL-8, NRC31, CCL24, CCR6	Lower transcription of IL-8 in MDD ad BPD patients as compared with controls. MDD patients exhibited decreased transcription of NRC31 relative to control subjects. Higher transcription of CCL24 consistently differentiated MDD patients from control and BPD subjects. Lower transcription of CCR6 consistently differentiated MDD patients from controls.	Peripheral blood
Rizavi et al. 2016^23^	30 MDD patients 31 healthy controls	RT-qPCR	IL-1b, IL-6, TNF-a, TNFR1, TNFR2, IL-1R1, IL-1RA, IL-1R2, IL-6R, Gp130	Significantly increased mRNA levels of the pro-inflammatory cytokines IL-1b, IL-6, TNF-a, their receptors, TNFR1, TNFR2, IL-1R1 and the antagonist IL-1RA were in the lymphocytes of MDD patients compared with controls. No significant differences in the lymphocyte mRNA levels of IL-1R2, IL-6R and Gp130 were observed between MDD patients and controls.	Peripheral blood leukocytes
Morrison et al. 2019^48^	25 MDD patients 12 PTSD patients 13 healthy controls	RT-qPCR	IL-1, IL-1B, IL-6, IL-8, IL-10, IL-13, IL-15	Significant decreases in gene expression of IL-1A in PTSD and depression cases relative to controls.	Dorsolateral prefrontal cortex (Brodmann Area 9/46)
Carvalho et al. 2014^49^	47 medication-free melancholic MDD patients 42 healthy controls	RT-qPCR	47 inflammation-related genes	34 monocyte inflammatory-related genes were significantly upregulated and 2 were significantly downregulated in MDD patients as compared to controls, the latter including the gene for the active GRα in particular in those with a high HAMD.	Monocytes

*MDD* major depressive disorder, *PCR* polymerase chain reaction, *DD* depressive disorder; *BPD* bipolar disorder, *rDD* recurrent major depression, *SSD* subsyndromal symptomatic depression, *RT-qPCR* reverse transcription (RT) quantitative PCR, *PTSD* post-traumatic stress disorder.

In contrast, Spindola et al.^26^ have investigated MDD in childhood and adolescence, analysing the mRNA expression of 12 genes including some inflammation-related genes. Interestingly, TNF, TNFR1 and IL-1b were expressed at significantly lower levels in the MDD group when compared with healthy controls suggesting that the regulation of inflammatory response might play a key role in early MDD pathophysiology. However, it has been proposed that findings in adults can differ from those in children^27^. In fact, factors such as traumatic events, abuse of alcohol and smoking identified in adulthood but not in childhood could affect MDD in adults.

Of course, the activation of the immune system observed in patients with MDD is not limited to changes in cytokines production. In fact, it has been postulated that oxidative stress, a trigger of inflammation, has an important role in the pathogenesis and neuroprogression of MDD^28^. In physiological conditions, multiple defence systems are involved in protecting cells from damage by reactive oxygen species (ROS). The main antioxidative enzymes (AOEs) include copper-zinc and manganese superoxide dismutase (CuZnSOD and MnSOD, respectively), catalase (CAT), glutathione peroxidase (GPx) and glutathione reductase (GLR)^29,30^. Antioxidant protection is tightly regulated by redox-sensitive transcriptional factors such as the nuclear factor (erythroid-derived 2)-like 2 (Nrf2)^31,32^ and NF-κB^33,34^. In this regard, Lukic et al.^35^ provided evidence that MDD is characterized by an upregulation of redox-sensitive transcriptional factors (Nrf2 and NF-κB) and AOEs (MnSOD, CuZnSOD and CAT), indicating a pro-oxidative state in the PBMC of MDD patients. Specifically, they found higher mRNA levels of Nrf2 and its regulator Keap1, as well as NF-κB in the cytoplasm of PBMC of depressed patients as compared to controls. This state was further reflected by increased levels of MnSOD, CuZnSOD and CAT proteins and by the lack of correlation between MnSOD and CAT, which, according to the authors’ hypothesis, could indicate impaired oxidative detoxification capacity in MDD patients. Moreover, the authors found a positive correlation between increased levels of MnSOD, CuZnSOD and CAT in MDD patients and the levels of Nrf2, while increased levels of SODs were also positively related to NF-κB. These findings suggest that alterations in antioxidative defence systems lead to an alteration in the pro-inflammatory signalling found in MDD.

Recently, it has also been reported that the neurotransmitter serotonin (5-HT) can regulate the immune system. Peripheral 5-HT is a potent immune modulator and affects immune cells via its receptors and the recently identified process of serotonylation, an independent mechanism by which serotonin leads to the activation of intracellular processes^36^. Based on this, Amidfar et al.^37^ measured the relative expression levels of 5-HT2A and 5-HT3A receptors in PBMCs of patients with MDD, and found that depressed patients have higher 5-HTR2A mRNA levels than healthy subjects.

Finally, in the Biodep study^38^ we have recently shown that drug-free and treatment-resistant depressed patients not only have higher pro-inflammatory cytokines/chemokines, but we have also shown an increased expression of the P2X purinoceptor 7 (P2RX7). P2RX7 has a crucial role in the activation of the inflammatory processes and it is ubiquitously expressed among cells of the immune system, including microglia cells^39^. Additionally, its expression has been identified in neuronal cells, where it can regulate the function of different neurotransmitters relevant to MDD^40^.

Overall, these studies have shown a positive correlation between an upregulated expression of pro-inflammatory molecules and MDD, suggesting that inflammation is one of the key factors involved in the pathogenesis and progression of MDD. Moreover, these studies suggest the utility of inflammation-related gene expression levels as biomarkers for MDD treatment response.

## Neuroplasticity

In addition to increased inflammatory levels, to date, several studies have demonstrated an impairment of neuroplasticity in MDD^41,42^. For example, alterations in synaptic and morphological plasticity have been reported in patients with MDD^43–45^. Numerous studies have also tried to understand the intracellular mechanisms underlying these alterations and their role in MDD (see Table 2). Evidence indicates that multiple neurotrophic/growth factors, such as brain-derived neurotrophic factor (BDNF) and glial cell-line-derived neurotrophic factor (GDNF) play a key role in neural plasticity^46,47^. BDNF is, in fact, involved in proliferation, migration, differentiation and survival of neurons in humans^48^. This finding has been confirmed by Hong et al.^49^, who examined the mRNA levels of BDNF and the mitogen-activated protein kinase 1/2 (MEK1/2), an immediate activator of the MEK–ERK pathway mediated by BDNF, in the leukocytes of MDD patients and healthy controls. Interestingly, the authors have shown decreased mRNA levels of BDNF and MEK1 in depressed patients as compared with controls, supporting the involvement of BDNF and MEK1 in the pathogenesis of MDD.

**Table 2:** Studies examining alterations in the expression levels of neuroplasticity-related genes.

Citation	Sample	Methods	Gene	Main findings	Tissue
Fuchsova et al. 2015^61^	25 MDD suicide, 25 healthy control	RT-qPCR Western blot	GPM6A, GPM6B, CORO1A, GIT1, CAMK2A, PLP1	Significant correlations among the expression levels of GPM6A, GPM6B, CORO1A, GIT1 and CAMK2A, but not PLP1 in the hippocampus of control subjects. Decreased GPM6A mRNA levels in the hippocampus of the depressed group as compared with controls. No significant differences were observed between the control and depressed groups for any of the members in the PFC. Decreased BDNF mRNA levels in the depressed subjects compared with controls in both the PCF and the hippocampus. Lower mRNA levels of CORO1A, GIT1 and CAMK2A in the hippocampus of suicides compared with controls.	Postmortem brain tissues
Mossakowska-Wójcik et al. 2017^65^	170 MDD patients 90 healthy controls	RT-qPCR	TCF4	Decreased TCF4 expression at the mRNA and protein levels in patients versus healthy individuals.	Peripheral blood
Iacob et al. 2013^69^	23 females with medication refractory DD: 13 with MDD, 10 with BPD. 19 healthy controls	RT-qPCR	APP, NR3G1	Only BPD patients showed increased APP and NR3G1 expression.	Peripheral blood leukocytes
Bobińska et al. 2017^67^	186 MRD patients 105 healthy controls	RT-qPCR	Neuropsin (NP)	Higher Human NP mRNA level in patients with depression than in the control group.	Peripheral blood
Berent et al. 2014^70^	38 MDD patients 38 healthy controls	RT-qPCR ELISA	VEGFA	Higher VEGFA mRNA and protein expression levels in MDD patients than in controls.	Peripheral blood
Cattaneo et al. 2013^25^	811 MDD patients: 296 men and 514 women 34 healthy controls: 19 males and 15 females	RT-qPCR	BDNF, p11, VGF	Lower mRNA levels of BDNF, p11 and VGF in MDD patients compared with controls.	Peripheral blood
Bhandage et al. 2017^71^	16 men 19 non-pregnant women 40 pregnant women: 25 healthy and 15 depressed	RT-qPCR	GluA1, GluA3, GluA4, GluK2, GluK4, GluK5, GluN2D, GluN1, GluN2C, GluD1, GluD2, GluN3A	Higher expression levels of GluA3 in men compared with pregnant women and in non-pregnant women compared with depressed pregnant women. Higher GluK4 expression levels in men and non-pregnant women than in pregnant women. High expression level of GluN2D in the PBMCs from the healthy pregnant women that were not observed either in depressed pregnant women, in non-pregnant women or men. The GluD1 expression level was higher in non-pregnant women as compared to men whereas the expression was more similar in men and pregnant women.	PBMC
Bobińska et al. 2016^72^	139 MDD patients 95 healthy controls	RT-qPCR	MMP-2, MMP-9, TIMP-2	Decreased expression of MMP-2, MMP-9 and TIMP-2 genes on both mRNA and protein levels in depression when compared with.	Peripheral blood
Guo et al. 2015^73^	50 first episode SSD 20 MDD patients 50 healthy controls	RT-qPCR	PRCKB1	PRCKB1 gene expression was downregulated in SSD patients, and a more dramatic downregulation in MDD patients than control.	PBMC
Hong et al. 2015^49^	50 MDD patients: 26 with treatment-resistant depression 24 with treatment-responsive depression 48 healthy controls	RT-qPCR	BDNF, MEK1	BDNF and MEK1 mRNA levels were significantly reduced in patients with MDD when compared with healthy controls, as well as among treatment-resistant depressive patients as compared with treatment-responsive depressive patients.	Peripheral blood leukocytes
Shibata et al. 2013^58^	44 BDP patients: 32 Remission and 12 Depressed 59 MDD patients:39 Remission and 20 Depressed 28 healthy controls	RT-qPCR	HIF-1, VEGF, GLUT1, PGK1, PFKFB3, LDHA	Increased expression of HIF- 1α and HIF-1β mRNA, VEGF and PFKFB3 in both MDD and BPD patients in a depressive state compared with healthy control subjects. mRNA expression levels of GLUT1, PGK1 and LDHA were increased in MDD patients in a depressive state compared with healthy control subjects. Increased expression of HIF-1α and LDHA mRNA in MDD patients in a remissive state, whereas the mRNA expression levels of other genes in a remissive state were comparable to those in healthy control subjects.	Peripheral blood

*MDD* major depressive disorder, *PCR* polymerase chain reaction, *DD* depressive disorder, *BPD* bipolar disorder, *rDD* recurrent major depression, *SSD* subsyndromal symptomatic depression, *RT-qPCR* Reverse transcription (RT) quantitative PCR, *PTSD* post-traumatic stress disorder.

Furthermore, vascular endothelial growth factor (VEGF), a neurotrophic and an angiogenic growth factor, has been implicated in different physiological processes such as angiogenesis, neuroprotection, neuronal survival, regeneration, growth, differentiation and axonal outgrowth^50–53^. Different studies have proposed that changes in VEGF expression levels can be linked to mood disorders, including MDD^54,55^.

A well-known oxygen-sensitive transcriptional activator of VEGF, the hypoxia inducible factor-1 (HIF-1), is induced by hypoxia, ischemia and by the activation of the expression of different genes such as VEGF, erythropoietin (EPO), glucose transporter-1,3 (GLUT1,3), lactate dehydrogenase-A (LDHA), phosphoglycerate kinase 1 (PGK1), 6-phosphofructo-2-kinase/fructose-2,6-biphosphatase-3 (PFKFB3), insulin-like growth factor-2 (IGF-2) and BCL2/adenovirus E1B 19 kDa interacting protein 3 (BNip3). Moreover, it contributes to angiogenesis, erythropoiesis, glucose metabolism, cell proliferation/survival and apoptosis^56,57^. According to this background, Shibata et al.^58^ investigated the mRNA expression levels of HIF-1 (α and β) and its target genes (VEGF, GLUT1, PGK1, PFKFB3 and LDHA) in peripheral white blood cells of patients affected by MDD and bipolar disorder (BPD). The authors found increased expression levels of HIF-1, VEGF, PFKFB3, GLUT1, PGK1 and LDHA in MDD subjects as compared to the control group.

Moreover, the neuronal membrane glycoprotein M6a (GPM6A), a member of the myelin proteolipid protein (PLP/DM20) family, plays an important role in stress response in different animal models^59,60^. Based on this notion, Fuchsova et al.^61^ hypothesized that alterations in the expression of the stress responsive neuroplasticity-related genes, such as the members of the PLP family, could be involved in the aetiology of MDD. They demonstrated that, GPM6A mRNA levels were significantly reduced in the hippocampus of depressed suicides. Conversely, GPM6B, but not PLP1, was downregulated. All these findings suggest that changes in the balance between mRNA levels of all the studied genes could lead to significant alterations in the neuronal connectivity causing pathological behaviours. According to the authors, these findings suggest that reduced GPM6B expression could contribute to oligodendrocyte misfunction linked with MDD.

Several studies have also suggested that the Transcription factor 4 (TCF4) gene is involved in the early differentiation of neurons, is related to memory efficiency^62^, and affects the immune response of the brain^63,64^. Mossakowska-Wójcik et al.^65^ analysed the mRNA and protein levels of TCF4 in blood of MDD patients and healthy subjects. TCF4 expression at both the mRNA and protein level was decreased in patients with MDD when compared with controls, suggesting that reduced mRNA and protein levels of the TCF4 gene might result in the worsening of cognitive functions, which could alter the development or course of MDD.

Furthermore, in 2012 Ziemiańska et al. showed that neuropsin (NP), a kallikrein gene-related endoprotease, has an important role in neuroplasticity processes, including intracellular signal cascades and regulation of gene expression that are involved in long-term synaptic plasticity^66^. In this regard, Bobińska et al.^67^ have compared the gene expression levels of NP gene in peripheral blood samples of a group of MDD patients and healthy subjects, showing that the expression levels of the human NP gene were significantly higher in MDD patients than in controls. According to the authors, a possible explanation of these results could be the young age of the examined individuals in both groups, in fact other studies have shown that NP expression levels gradually decrease in the cerebral cortex during ageing^68^.

Altogether, the presented studies have shown that a dysregulation of neurotrophic/growth factor systems such as BDNF and VEGF as well as of other genes involved in the regulation of neuroplasticity can underlie the development of cognitive impairment, often observed in MDD.

## Neurotransmitters

Research over the years has attempted to define the relationships between specific neurotransmitters in the brain and specific symptoms of MDD. Indeed, it has been proposed that different neurotransmitters may regulate different brain functions, neurochemical mechanisms, and subsequently, specific antidepressant drugs could target symptom-specific neurotransmitters^69^. MDD has been widely linked to imbalances in the brain with regard to the neurotransmitters serotonin, norepinephrine and dopamine and, recently, another neurotransmitter, glutamate, has been also implicated in MDD.

Interestingly, it has been shown that the release of some neurotransmitters, including the release of noradrenaline, serotonin, GABA, glutamate, and dopamine, is facilitated by the activated a-7 nicotinic acetylcholine receptor (a7 nAChR) via the increased permeability to cations, including Ca(2+)^70^. A7 nAChR is coded by the Cholinergic Receptor Nicotinic Alpha 7 Subunit (CHRNA7) gene, which is partially duplicated by a chimeric gene, CHRFAM7A. On these bases, Kunii et al.^71^ (Table 3) have investigated the expression of CHRNA7 and CHRFAM7A in the dorsolateral prefrontal cortex in a large cohort of patients with schizophrenia, BPD and MDD. They found that the expression levels of CHRNA7 were significantly increased in MDD patients as compared with all other groups. Similarly, the expression of CHRFAM7A was significantly elevated in all diagnostic groups, especially in the MDD group, as compared with the healthy group and the ratio of CHRFAM7A/CHRNA7 levels was significantly different between the diagnostic groups, suggesting an aberrant function of nAChRs in mental illnesses.

**Table 3:** Studies examining alterations in the expression levels of neurotransmission-related genes.

Citation	Sample	Methods	Gene	Main findings	Tissue
Kunii et al. 2015^71^	176 schizophrenic patients 61 BPD patients 138 MDD patients 326 healthy controls	RT-qPCR	CHRFAM7A, CHRNA7	Increased expression levels of CHRNA7 mRNA in MDD patients compared with all other groups. Expression of CHRFAM7A was significantly elevated in all diagnostic groups compared with the control group.	Postmortem brain tissues
Gray et al. 2015^80^	53 MMD patients: 26 males and 27 females 34 MDD suicide and 19 MDD non-suicide 32 healthy controls: 19 males and 13 females	RT-qPCR	GluR genes	The majority of the 21 GluR genes that were tested showed higher levels of expression in the MDD subjects relative to controls. The greatest effects were detected in the female groups.	Postmortem brain tissues
Chandley et al. 2013^86^	19 MDD patients 20 healthy control	RT-qPCR immunostaining	SLC1A3, SLC1A2, GLUL, GFAP	Astrocytes, but not oligodendrocytes, demonstrated robust reductions in the expression of SLC1A3 and SLC1A2, whereas GLUL expression was unchanged in MDD patients when compared with controls. GFAP expression was lower in astrocytes, and we confirmed reduced GFAP protein in the LC using immunostaining methods.	Postmortem brain tissues
Chandley et al. 2014^81^	18 MDD patients 18 healthy controls	RT-qPCR	GRIN1, GRIN2A, GRIN2B, GRIN2C, GRIN2D, GRIN3A, GRIN3B, GRIA1, GRIA2, GRIA4, GRIK1, GRIK3, GRIK5, GRM4, GRM5, GRM8	MDD subjects exhibited significantly higher expression levels of the NMDA receptor subunit genes, GRIN2B and GRIN2C and the metabotropic receptor genes, GRM4 and GRM5, in LC neurons.	Postmortem brain tissues
Oh et al. 2014^88^	15 MDD patients: 7 with suicide, 8 without suicide 15 healthy controls	Reanalysis of existing postmortem data, found in the Stanley neuropathology consortium integrative database, a web-based tool that integrates datasets from the same postmortem brain samples.	GAD1, SLC1A1, SLC1A2, SLC1A3	The mean GAD1 mRNA was almost all slightly lower in the subjects with MDD as compared with the control group. While SLC1A1-3 mRNA expression was generally lower in the subjects with MDD as compared to the controls in each sub-region, it was only in the white matter (BA46) that SLC1A2 mRNA was significantly lower in the subjects with MDD as compared to the normal control group.	Postmortem brain tissues

*MDD* major depressive disorder, *PCR* polymerase chain reaction, *RT-qPCR* reverse transcription (RT) quantitative PCR, *BPD* bipolar disorder.

Moreover, norepinephrine has a role in the recognition and response to stressful situations, and it has been suggested that an aberrant norepinephrinergic system could lead to an increased vulnerability to MDD^72^. Dopamine plays an important role in regulating our drive to seek out rewards, as well as our ability to obtain a sense of pleasure. Low dopamine levels could help to explain why people suffering from MDD do not show the same sense of pleasure^73^. 5-HT is a monoamine involved in a number of physiological processes, and MDD appears, in part, to be a result of diminished activity of the serotonin system^69^. 5-HT is both a neurotransmitter and a neuromodulator that regulates different pathophysiological aspects of MDD, including mood, sleep, energy balance and immunity^74–76^.

While the role of these three neurotransmitters (norepinephrine, dopamine and 5-HT) in MDD has been studying for many years^77^, the implication of glutamate in this psychiatric disorder has been recently discovered. Indeed, a growing body of data shows that abnormalities of the glutamate system lead to altered behaviours that correlate with psychiatric disorders, including MDD^78^. Glutamate is an excitatory neurotransmitter that is widely distributed in the brain, exerting its effects through the stimulation of several glutamate receptor (GluR) subtypes. These include the 2-amino-3-(3-hydroxy-5-methyl-iso- xazol-4-yl) propanoic acid (AMPA), N-methyl-D-aspartate (NMDA), kainate (KAR) and metabotropic (mGluR) receptors^79^. Four studies have mainly analysed mRNA expression in postmortem brain tissues of glutamate receptors and transporters (Table 3).

For instance, Gray et al.^80^ have tested the hypothesis that GluR gene expression is altered in the dorsolateral prefrontal cortex (DLPFC) in MDD in a large cohort of postmortem subjects from three diagnostic groups: MDD suicide, MDD non-suicide and a group of controls with no history of psychiatric disorders. They have reported higher expression levels of a number of GluR genes in the DLPFC of MDD patients as compared to controls. In particular, they have found higher expression levels of GRIN1, GRIN2A-D, GRIA2-4, GRIK1-2, GRM1, GRM4, GRM5 and GRM7 in female patients as compared to male patients with MDD. In contrast, GRM5 expression levels were lower in male MDD patients than in male controls and, finally, in all sample (both male and female) when MDD suicides were compared with MDD non-suicides, GRIN2B, GRIK3 and GRM2 were expressed at higher levels in the suicide subjects. Taken together, these data indicate that a disruption of the glutamate system occurs in the DLPFC of patients with MDD, above all in those who completed suicide. According to the authors, this disruption may be more severe in female patients.

In addition, because of several studies indicate that the locus coeruleus (LC) has a major role in the origin of clinical MDD and possibly suicide, Chandley et al.^81^ examined the gene expression levels of glutamate receptors, NMDA and AMPA in postmortem noradrenergic LC neurons from subjects with MDD (most of which died by suicide) and matched to healthy controls. They evaluated the expression of all NMDA receptor subunit genes in the LC and for the remaining glutamate receptor genes, including the AMPA, kainate and metabotropic glutamate receptors, examining only those that demonstrated measurable gene expression in the mouse LC, according to the Allen Brain Atlas, an online publicly available resource that integrates gene expression and connectivity data with neuroanatomical information for the mouse, human and non-human primate^82,83^. They found elevated expression levels of genes encoding specific ionotropic NMDA receptor subunits and specific metabotropic receptors in both MDD and control subjects. Specifically, the authors found highly expressed GRIN1 subunit, moderate gene expression levels of GRIN2A, GRIN2B, GRIN2D subunits and lower levels of GRIN2C and GRIN3A subunits. The functional NMDA receptor complex is made of a glycine binding NR1 subunit combined with at least one of the other glutamate binding NR2 or NR3 subunits. Although the NMDA receptor complex is permeable to both potassium and calcium, calcium is essential in activating the PI3K and CREB cell-signalling pathways that distinguish the NMDA family of receptor signalling from the other ionotropic glutamate receptors^84,85^. This is particularly intriguing since, in the same work, Chandley and colleagues^81^ observed elevations in NMDA receptor subunit gene expression in MDD patients when compared to controls, but no expression changes in the moderately expressed GluA1 receptor (GRIA1) or the highly expressed GluA2 (GRIA2) and GluA4 (GRIA4) of the AMPA ionotropic family, nor in any of the receptor subunits (GRIK1, GRIK3 and GRIK5) from the kainate ionotropic class of receptors. Moreover, they have found an increase in expression levels of two metabotropic glutamate receptor genes (GRM5, GRM4) in LC neurons from MDD subjects in comparison to normal control subjects.

Earlier, in 2013, the same authors^86^ examined the expression of three glutamate-related genes (two glutamate transporters, SLC1A3 and SLC1A2, and an encoding glutamine synthase GLUL) concentrated in glia, and of a glia gene (GFAP) in postmortem tissues from men with MDD and from matched healthy controls. They found evidence of astrocyte dysfunctions in the LC region in individuals with MDD, which included reduced expression levels of SLC1A3, SLC1A2 and GFAP, together with lower GFAP protein levels, and reduced density of GFAP-positive astrocytes. This study provided a direct evidence of astrocyte pathology in LC, indicating that glia cell abnormalities reported in more superior/rostral brain regions^43,87^ extend to the brainstem and may contribute to the pathology of the monoamine systems in MDD. Similarly, Oh et al.^88^ studied the role of the glutamate transporters (SLC1A2 and SLC1A3) in the dorsolateral prefrontal cortex of MDD subjects. Using data from the Stanley neuropathology consortium integrative database (SNCID^89^), they analysed the mRNA levels of the gamma-aminobutyric acid-synthesizing enzyme (GAD1) and investigated a possible linkage between changes in SLC1A2 and GAD1 expression levels. They observed that the expression levels of GAD1 and SLC1A2 were lower in the DLPFC of subjects with MDD as compared to controls and, that GAD1 mRNA levels were significantly associated with SLC1A2 mRNA expression levels in the same area in the group of MDD patients.

All the above-mentioned studies have demonstrated the involvement of several neurotransmitters in the pathogenesis of MDD. Particularly, they not only have consolidated the role of serotonin, dopamine and norepinephrine, but also shown abnormalities of the glutamate system. In fact, these studies have observed that the pathophysiology of MDD is associated with dysfunctions in the glutamatergic system, and with alterations in the mechanisms regulating the clearance and metabolism of glutamate in brain areas mediating cognitive–emotional behaviours.

## Stress-related mechanisms

Stress and/or trauma are associated with dramatic increases in the risk of developing depressive disorders^90^. The stress response system is linking the CNS and the endocrine system and it allows responding to short-term and long-term stressors. The key neuroendocrine component of this response to stress is the HPA axis, which acts as an interface between cognitive and non-cognitive stressors processed in the CNS and in the peripheral endocrine response system^91^. To understand the mechanisms of stress response, several studies have assessed the mRNA levels of genes involved in the stress response in patients with MDD (see Table 4). It is well known that the glucocorticoid receptor (GR) plays a crucial role in mediating the negative feedback regulation of the HPA axis^92,93^ and, recently, several studies have investigated the GR expression levels and functionality in patients with MDD. To this purpose, Roy et al.^94^ have studied the mRNA levels of stress-related genes, such as BDNF, Nuclear Receptor Subfamily 3 Group C Member 1 (NR3C1 or GR), FK506 Binding Protein 5 (FKBP5), Corticotropin Releasing Hormone Binding Protein (CRHBP), and Corticotropin Releasing Hormone Receptor 1 (CRHR1) in PBMC of MDD patients and their matched controls. NR3C1 encodes the GR, which can function both as a transcription factor that binds to glucocorticoid responsive elements (GRE) in the promoters of glucocorticoid responsive genes by activating their transcription, and as a regulator of other transcription factors. FKBP5 is a co-chaperone of hsp90, which regulates GR’s sensitivity, whereas BDNF expression is regulated by GR. The authors have found a reduction in the expression levels of most of the analysed genes, including BDNF, FKBP5 and NR3C1 in MDD patients as compared to controls, confirming that lower expression levels of these transcripts may induce a maladaptive response toward stressful stimuli, increasing the risk for MDD.

**Table 4:** Studies examining alterations in the expression levels of stress-related genes.

Citation	Sample	Methods	Gene	Main findings	Tissue
Cattaneo et al. 2020^38^	130 MDD patients: 36 Treatment-responsive 36 Drug-free 58 Treatment-resistant 40 healthy controls	RT-qPCR	FKBP5, GR, SGK1	Treatment-resistant and drug-free depressed patients had evidence of lower GR and higher FKBP5 mRNAs expression, Responsive patients were indistinguishable from controls.	Peripheral blood
Spindola et al. 2017^26^	20 MDD children and adolescents 49 participants without MDD with high levels of depressive symptoms (DS), 61 healthy controls	RT-qPCR	NR3C1	Decreased mRNA levels of NR3C1 in MDD group compared with controls and to DS group.	Peripheral blood
Anacker et al. 2013^97^	25 MDD patients: 7 drug-free, 18 drugs naïve 14 healthy controls	Western blot RT-qPCR	SGK1	Depressed patients had significantly higher SGK1 mRNA levels when compared with controls.	Peripheral blood
Roy et al. 2017^94^	14 MDD patients: 20 healthy controls	RT-qPCR MeDIP analysis	BDNF, FKBP5, CRHBP, NR3C1	Increase in DNA methylation of stress-related genes BDNF, NR3C1, FKBP5 and CRHBP in MDD patients compared with healthy controls.	PBMC
Iacob et al. 2013^95^	23 females with medication refractory DD: 13 with MDD, 10 with BPD. 19 healthy controls	RT-qPCR	OXTR, NFKB1, NR3C1	MDD patients showed increased expression levels of OXTR and confirmed a dysregulation in oxytocinergic signalling when compared with controls.	Peripheral blood leukocytes
Teyssier et al. 2012^98^	17 MDD patient 16 healthy controls	RT-qPCR	FOS, OGG1, STMN1, TERT, p16INK4a	OGG1, p16INK4a and STMN1 gene were significantly upregulated in the leucocytes of MDD patients.	Peripheral blood leukocytes

*MDD* major depressive disorder, *PCR* polymerase chain reaction, *DD* depressive disorder, *BPD* bipolar disorder, *RT-qPCR* reverse transcription (RT) quantitative PCR.

Similarly, Iacob et al.^95^ analysed the expression levels of glucocorticoid and mineralocorticoid receptors, respectively, NR3C1 and NR3C2, and also genes related to the glucocorticoid pathway as oxytocin prepropeptide encoding gene (OXT) and oxytocin receptor (OXTR). They observed that MDD patients showed increased expression levels of OXTR and confirmed deregulation in the oxytocinergic signalling, referring to signalling pathway proteins including oxytocin, oxytocin receptors and related regulatory factors.

Another important gene involved in the mediation of the glucocorticoid effects on brain function is a serine/threonine kinase (Serum/Glucocorticoid Regulated Kinase 1 (SGK1)), which plays a key role in the cellular response and neuronal functions, including adult hippocampal neurogenesis^96^. In fact, Anacker et al.^97^ found an increase in the SGK1 gene expression levels in the peripheral blood of drug-free depressed patients, identifying SGK1 as a key gene involved in the GR activation, which may be of particular relevance for stress-induced mental disorders, such as MDD.

To assess the hypothesis that stress is associated with MDD, Teyssier et al.^98^ measured the expression of a set of candidate biomarkers in peripheral blood leukocytes. These genes are FOS and DUSP1 (involved in the cell-signalling response to biopsychological stress), TERT, STMN1 and p16INK4a (biomarkers of telomere dysfunction and cellular senescence), and OGG1 (which catalyses the repair of oxidized 8-oxoguanine DNA base and is a sensible marker of oxidative stress). The OGG1, p16INK4a and STMN1 genes were significantly upregulated in the leucocytes of MDD patients when compared to controls, indicating an association between the upregulation of these transcripts and the increased risk of developing MDD.

Although overall it has been shown that depressed patients show altered expression levels of stress-related genes in peripheral blood samples, some of the previously mentioned studies highlighted also the presence of contrasting results that could be due to the patients’ pharmacological treatment. However, this should be better investigated in further studies.

## Treatment

Antidepressant therapy is an essential treatment for MDD, however, a substantial group of treated patients do not respond to the therapy, or suffer from severe side effects, such as gastrointestinal (GI) disturbances, anxiety, agitation and insomnia^99^. To date, different studies have been carried out to identify and validate biomarkers involved in the antidepressant treatment response (Table 5). This might open the door to personalized medication and, thus, might improve treatment efficacy and reduce side effects.

**Table 5:** Studies examining alterations in the expression levels of genes related to antidepressant treatment response.

Citation	Sample	Antidepressant treatment	Methods	Gene	Main findings	Tissue
Cattaneo et al. 2020^38^	130 MDD patients: 36 Treatment-responsive 36 Drug-free 58 Treatment-resistant 40 healthy controls	Different antidepressants–non-interventional study	RT-qPCR	IL-1b, IL-6, MIF, TNF-a, P2RX7, CCL2, CXCL12, AQP4, ISG15, STAT1, USP18, FKBP5, GR, SGK1	Evidence of increased inflammasome activation (higher pro-inflammatory cytokines/chemokines and P2RX7 mRNAs expression) in treatment-resistant and drug-free depressed patients compared with controls and responsive patients. Lower GR and higher FKBP5 mRNAs expression in treatment-resistant and drug-free depressed patients when compared to controls and responsive patients. Responsive patients were indistinguishable from controls, except for having lower CXCL12.	Peripheral blood
Breitfeld et al. 2017^103^	25 therapy-resistant patients 25 first-line therapy responders	Different antidepressants	RT-qPCR	WNT2B, FZD7, ABCB1	Significantly increased levels of genes WNT2B, FZD7 and ABCB1 in responder-derived cell lines when compared with controls, fold changes by SSRIs.	Lymphoblastoid cell lines
Shibata et al. 2013^58^	44 BDP patients: 32 Remission 12 Depressed 59 MDD patients: 39 Remission 20 Depressed 28 healthy controls	Different antidepressants	RT-qPCR	HIF-1, VEGF, GLUT1, PGK1, PFKFB3, LDHA	Increased expression of HIF-1α and HIF-1β mRNA, VEGF and PFKFB3 in both MDD and BPD patients in a depressive state compared to healthy control subjects. mRNA expression levels of GLUT1, PGK1 and LDHA were increased in MDD patients in a depressive state compared to healthy control subjects. Increased expression of HIF-1α and LDHA mRNA in MDD patients in a remissive state.	Peripheral blood
Belzeaux et al. 2014^109^	13 patients with severe major depressive episode 13 healthy controls	Imipramine	RT-qPCR	SLC6A4	Decrease of SLC6A4 mRNA expression in responder patients across a 30-week follow-up, while non-responder patients exhibited upregulated SLC6A4 mRNA.	PBMC
Cattaneo et al. 2013^25^	811 adult outpatients suffering from unipolar depression: 51 responders 23 non-responders 34 healthy controls:19 males 15 females	Escitalopram Nortriptyline	RT-qPCR	FKBP-4, FKBP5, GR, IL-1a, IL-1b, IL-4, IL-6, IL-7, IL-8, IL-10, TNF-a, MIF, BDNF, p11, VGF	Higher levels of IL-1b, IL-6, MIF, TNF-a and FKBP5, in depressed patients as compared with controls. Lower levels of IL-4, GR, BDNF, p11 and VGF in depressed patients as compared with controls. Antidepressant treatment significantly reduced FKBP5, IL-1b, MIF, TNF-a, IL-6 and VGF levels only in patients who responded to the treatment and increased GR mRNA levels and p11 levels. Antidepressant treatment increased BDNF expression more in the responders than in the non-responders.	Peripheral blood
Eyre et al. 2017^125^	56 depressed patients: 20 treated with vilazodone 25 treated with paroxetine	Vilazodone, paroxetine	RNA expression profiling assays	NF-κB, AP-1, cAMP	Reduced NF-κB, AP-1 and cAMP activity in the vilazodone group compared to the paroxetine group.	Peripheral blood leukocytes
Alcocer-Gómez et al. 2013^111^	40 MDD patients: 20 without treatments 20 treated	Amitriptyline	Western blot RT-qPCR	NLRP3, Caspase-1, IL-1b, IL-18, ROS, LPO	Increased gene expression of NLRP3 and caspase-1 in blood cells in non-treated patients as compared with treated patients. Increased serum levels of IL-1b and IL-18 in non-treated patients as compared with treated patients. Amitriptyline treatment reduced NLRP3 and caspase-1 gene expression, and IL-1b and IL-18 serum levels. Oxidative damage was higher in MDD patients treated with amitriptyline.	PBMC
Hong et al. 2014^49^	50 MDD patients:26 non-responders 24 responders 48 healthy controls	Different antidepressants	RT-qPCR	BDNF, MEK1	BDNF and MEK1 mRNA levels were significantly reduced in patients with MDD when compared with healthy controls, as well as among treatment-resistant depressive patients as compared with treatment-responsive depressive patients.	Peripheral blood leukocytes

*MDD* major depressive disorder, *PCR* polymerase chain reaction, *BPD* bipolar disorder, *RT-qPCR* reverse transcription (RT) quantitative PCR.

In order to provide evidence supporting a personalized medicine approach for the treatment of MDD, Cattaneo et al.^25^ analysed the blood mRNA expression levels of 15 candidate genes across three biological systems, such as the GR complex, inflammation and neuroplasticity that have been more consistently described as abnormal in MDD^100^. To this purpose, they examined a well-characterized group of MDD patients from the GENDEP study^101,102^, before and after 8 weeks of treatment with one of two pharmacologically different antidepressants: the selective serotonin reuptake inhibitor, escitalopram and the tricyclic noradrenaline reuptake inhibitor, nortryptline. Cattaneo and her team measured the transcriptional levels of the following genes: FKBP-4, FKBP5 and GR, for the GR complex; IL-1a, IL-1b, IL-4, IL-6, IL-7, IL-8, IL-10, MIF and TNF-a, for the inflammatory system; BDNF, p11 and VGF (non-acronymic), for neuroplasticity. Data showed a dissociation between genes that predict treatment response (‘predictors’) and genes that change longitudinally in patients who respond (‘targets’) to antidepressant treatment. Specifically, among the 15 genes, only higher levels of three inflammation-related genes, IL-1b, MIF and TNF-a, predict a lack of response to antidepressants, even if a successful antidepressant response is not associated with a reduction in the levels of these genes. In contrast, a successful antidepressant response is associated with a reduction in the levels of the inflammation-related gene, IL-6, and of the GR-associated gene, FKBP5, as well as with an increase in the neuroplasticity-associated genes, VGF and BDNF.

Following this study, our group has carried out the largest non-interventional study so far investigating candidate mRNA gene expression in depressed patients characterised by their current depressive symptoms and by their response to antidepressant treatment^38^. As previously mentioned, we have found that treatment-resistant and drug-free depressed patients have an increased inflammasome activation (higher pro-inflammatory cytokines/chemokines and P2RX7 mRNAs expression) and glucocorticoid resistance (lower GR and higher FKBP5 mRNAs expression); whereas responsive patients were alike controls except for having lower CXCL12.

According to the neurotrophic hypothesis of MDD, an association between effects on neuroplasticity and clinical response to antidepressant drug therapy has been suggested by several studies. For example, Breitfeld et al.^103^ have tried to identify a possible association between functional biomarkers related to neuroplasticity effects of antidepressants with treatment response and resistance in patient-derived lymphoblastoid cell lines (LCLs) from the STAR*D study. Specifically, they identified five potential biomarkers that have been associated with cell proliferative effects of antidepressants (ex vivo) or with LCL donor’s clinical response/remission in antidepressant drug therapy: transcription factor 7-like 2 (TCF7L2), frizzled class receptor 7 (FZD7), wingless-type MMTV integration site family member 2B (WNT2B), p-glycoprotein (ABCB1) and sulfotransferase 4A1 (SULT4A1). Interestingly, the most notable differences in the expression levels between responder- and treatment resistance-derived LCLs were observed for WNT2B, FZD7 and ABCB1. ABCB1 is the most studied member of the ATP-binding cassette (ABC) transporter family and it plays a key role in cellular detoxification and transmembrane transport across the blood–brain barrier. The allocrite spectrum includes neurotoxic agents (such as glucocorticoids, drugs and xenobiotics) and hence, ABCB1 has neuroprotective effects resulting in a possible increased response to antidepressants. WNT2B and FZD7 are elements of the canonical WNT signalling pathway regulating neurogenesis, synaptic plasticity and dendritic arborization^104^. While FZD7 inhibits the WNT signalling, WNT2B and chronic antidepressant treatment activate this pathway resulting in increased neurogenesis. Altogether these effects might be responsible for enhanced neuronal plasticity and likely for remission from MDD.

Moreover, the serotonin transporter has been linked to MDD in candidate gene studies and in gene-to-environment interaction studies, hence it plays a key role in MDD pathophysiology^105,106^. The serotonin transporter protein (SLC6A4) is the main target of many antidepressants, although the relationship between pathophysiology and therapeutic effects of antidepressants is still not clear^107,108^. Based on previous studies on SLC6A4 mRNA gene expression variation in peripheral tissues, Belzeaux et al.^109^ explored whether SLC6A4 mRNA could be a target biomarker of antidepressant treatment during a major depressive episode that varies between the baseline and the 30-week follow-up period in responder patients. Interestingly, decreased expression levels of SLC6A4 were observed in responder patients across a 30-week follow-up, whereas non-responder subjects showed increased mRNA levels of SLC6A4. Conversely, healthy controls exhibited a stable pattern of SLC6A4 mRNA expression across the 30-week follow-up period. These data support that the serotonin transporter protein, the main target of many antidepressants, could be a valid target biomarker in MDD patients for a personalized medicine approach.

As suggested by our results in responder and non-responder patients, gene expression variation of selected genes, monitored across a long period of time, could be informative of clinical evolution and potential relapses or recurrences.

Another interesting hypothesis of MDD suggests that the inflammasome is a central mediator by which psychological and physical stressors could contribute to the development of the disorder^110^. In this regard, the study performed by Alcocer-Gómez et al.^111^ examined this hypothesis to determine whether NLRP3 inflammasome could be activated in PBMC from MDD patients and to shed light on the implication of mitochondrial oxidative stress. Furthermore, they studied the effects of amitriptyline, a tricyclic antidepressant drug, on NLRP3 inflammasome activation. The authors found that MDD patients showed reduced serum levels of IL-1b and IL-18, and a significant reduction in NLRP3 and caspase-1 activation. Moreover, they observed that the association between the Beck’s Depression Inventory (BDI) scores and IL-1b and IL-18 serum levels was reduced when controlling for antidepressant treatment, suggesting that antidepressants can modulate the inflammation levels.

Another promising candidate in the field of pharmacological treatment options regarding MDD is represented by the mitochondrial translocator protein (TSPO), a 5-helical transmembrane protein located in the outer mitochondrial membrane^112^. It plays an important role in neurosteroid synthesis and in systemic endocrine regulation, with implications in the pathophysiology of immune, inflammatory, neurodegenerative, neoplastic and psychiatric diseases^113^. Interestingly, Sarubin et al.^114^ investigated the effects of antidepressant treatment on TSPO expression levels in platelets obtained from 37 patients suffering from MDD, analysing TSPO levels in depressed patients before and after 6 weeks of antidepressant treatment. A significant change in TSPO levels over 6 weeks of treatment was observed within the complete sample of MDD patients. Interestingly, responders showed a greater reduction in TSPO levels as compared to non-responders. These results are in contrast with the hypothesis of the authors, expecting to find increased TSPO levels during antidepressant therapy along with a decrease in depressive symptoms. Therefore, they concluded that TSPO expression in platelets cannot be considered an appropriate biomarker for the analysis on the course of MDD.

Overall, the above studies have shown that patients who responded to the antidepressant therapy had restored levels of inflammation-related genes, such as IL-6 and IL-1b, of stress-related genes, including FKBP5, as well as of neuroplasticity-associated genes, such as VGF and BDNF.

## Whole-genome transcriptome assays

High-throughput technologies such as microarrays allow to explore the expression levels of the whole genome and the identification of changes in gene expression by using a hypothesis-free approach. In the last decade, several studies have used these technologies to identify gene expression differences related to MDD (Table 6). Together with the hypothesis-driven approach, mainly based on the analysis of candidate genes expression levels, transcriptomics studies can allow the identification of new biomarkers associated with MDD that can help the development of novel intervention strategies and the introduction of personalized medicine.

**Table 6:** Studies examining alterations in the expression levels of genes associated with depression using transcriptomics techniques.

Citation	Sample	Methods	Main findings	Tissue
Leday et al. 2017^115^	GSK-HiTDiP: 113 MDD patients57 healthy control Janssen–BRC: 94 MDD patients 100 healthy control	Microarray	A total of 165 genes were differentially expressed in both studies with concordant direction of fold change. The 90 genes overexpressed in MDD were significantly enriched for immune response to infection, were concentrated in a module of the gene co-expression network associated with innate immunity and included clusters of genes with correlated expression in monocytes, monocyte-derived dendritic cells, and neutrophils. The 75 genes downregulated in MDD were associated with the adaptive immune response and included clusters of genes with correlated expression in T cells, natural killer cells and erythroblasts.	Peripheral blood
Malki et al. 2014^116^	11 MDD patients 15 healthy controls	Microarray	Out of a total of 15 genes, VAMP-2 is significantly downregulated in MDD patients when compared with controls.	Postmortem prefrontal cortex
Mostafavi et al. 2014^117^	467 patients with rDD 459 healthy controls	HTSeq	Significant association was observed between MDD and the expression of genes involved in IFN α/β signalling pathway.	Peripheral blood
Jansen et al. 2016^118^	882 current MDD 635 remitted MDD 331 healthy controls	Microarray	Genes associated with MDD were enriched for IL-6 signalling and NK cell pathways. Thirteen gene expression clusters with specific clusters enriched for genes involved in NK cell activation (downregulated in current MDD,) and IL-6 pathways (upregulated in current MDD) were identified.	Peripheral blood
Yamagata et al. 2017^119^	23 MDD patients: 10 depressed state 13 remitted state 30 healthy controls	Microarray	SLC35A3, HIST1H2AL, YEATS4, ERLIN2 and PLPP5 were downregulated in patients with MDD when compared with controls.	Leukocytes
Duric et al. 2013^126^	21 MDD patients 18 healthy controls	Microarray	Downregulation of several pre- and post-synaptic genes in MDD subjects when compared with controls. Decreases in expression of MAP1A, MAP1B, MAP2 and MAPT genes and of AMPA receptors genes, specifically GLUR1 and GLUR3 subunits observed in both the DG and CA1 of subjects with MDD when compared with controls.	Postmortem brain tissues
Fan et al. 2014^120^	91 MDD patients 46 healthy control	Microarray	26 miRNAs were identified with significantly different expression levels in MDD patients compared with controls. Of these, 21 miRNAs were upregulated, and 5 others were downregulated in MDD patients compared with controls. With the exception of miRNA-338, the expressions of the other 9 miRNAs conformed to microarray assay results, among which 5 miRNAs (miRNA-26b, miRNA-1972, miRNA-4485, miRNA-4498 and miRNA-4743) were upregulated with a significant difference in MDD patients compared with controls.	PBMC
Hennings et al. 2015^127^	24 male MDD patients: 12 responders 12 non-responders 142 unipolar depressed patients:80 responders 62 non-responders	Microarray	127 transcripts were significantly associated with treatment response. Lower expression of retinoid-related orphan receptor alpha (*RORa*), germinal center expressed transcript 2 (*GCET2*) and chitinase 3-like protein 2 (*CHI3L2*) on admission were associated with beneficial treatment response. In addition, leukocyte-specific protein 1 (*LSP1*) significantly decreased after 5 weeks of treatment in responders.	Peripheral blood
Woo et al. 2018^121^	38 Korean patients with MDD 14 healthy individuals	Microarray	CD58, CXCL8, EGF, TARP, TNFSF4, ZNF583 and ZNF587. CXCL8, EGF, and TNFSF4 genes were downregulated in MDD patients, whereas the other genes were upregulated in MDD patients.	Peripheral blood
Watanabe et al. 2015^122^	Pilot study: 25 drug-naive MDD patients 25 healthy controls Subsequent replication study: 20 drug-naive MDD patients 18 healthy controls	custom-made PCR array plates	Among 40 candidate genes, the expression levels of seven genes (PDGFC, SLC6A4, PDLIM5, ARHGAP24, PRNP, HDAC5 and IL-1R2) significantly differed between MDD and control samples in the pilot study.Ultimately, five genes (PDGFC, SLC6A4, ARHGAP24, PRNP and HDAC5) whose expression best differentiated between MDD patients and controls were selected for a multi-assay diagnostic test.	Peripheral blood
Hepgul et al. 2016^128^	20 MDD patients with chronic HCV infection due to commence combination antiviral therapy with IFN-α and ribavirin for at least 24 weeks 38 healthy controls	Microarray	506 genes were modulated only in patients who developed depression and 70 genes were modulated only in patients who did not develop depression. Pathway analysis of 506 genes modulated only in patients who developed depression were identified 65 pathways, including those related to inflammation (IL-1, IL-6 and IL-8 signalling, GR signalling, triggering receptor expressed on myeloid cells 1 signalling and NF-κB signalling), neuroplasticity (extracellular signal-regulated kinase 5 (ERK5) signalling and axonal guidance signalling), and oxidative stress (NRF2-mediated oxidative stress response, p53 signalling and production of nitric oxide and reactive oxygen species in macrophages).	Peripheral blood
Eyre et al. 2016^123^	35 MDD patients: 24 remitters 11 non-remitters randomized to methylphenidate and citalopram, citalopram and placebo or methylphenidate and placebo	RNA expression profiling assays	18 genes had higher expression levels in the group of early remitters versus non-remitters.	PBMC
Ju et al. 2019^129^	211 MDD patients treated with escitalopram 112 healthy controls	Array-Based Gene Expression Analysis	Increased mRNA expression of CHN2 and JAK2 in the non-responders group.	Peripheral blood

*MDD* major depressive disorder, *PCR* polymerase chain reaction, *HCV* hepatitis C virus, *rDD* recurrent major depression.

Recently, Hepgul et al.^115^ investigated whether gene expression changes in peripheral blood of patients with Hepatitis C at the baseline are associated with the future development of IFN-α-induced MDD (before IFN-α administration) and identified longitudinal changes in gene expression from baseline to treatment week (TW) 4 and TW24 following IFN-α treatment, in those subjects who did or did not develop MDD. Specifically, at the baseline, 73 genes were differentially expressed between patients who later developed MDD and those who did not. At TW4, 592 genes, primarily IFN-α-responsive genes, were significantly modulated in the whole sample; most of these genes were modulated only in patients who developed MDD, with an enhancement in inflammation-, neuroplasticity- and oxidative stress-related genes. Similar results were observed at TW24. These data clearly indicate that patients who develop IFN-α-induced MDD have an augmented biological sensitivity to IFN-α. Beyond the IFN-α treatment, the identified transcriptomics signature could be used as a biomarker for the early identification of individuals at high risk of developing MDD or to generate molecular targets for the discovery of new therapeutic strategies in MDD.

Another microarray study carried by Hennings et al.^116^, performed on peripheral blood samples collected at the admission and after 2 and 5 weeks of treatment from MDD male patients remitters and non-responders, identified 127 transcripts significantly associated with the treatment response. The authors also analysed these transcripts in an independent replication sample of 142 depressed in patients confirming that lower expression of retinoid-related orphan receptor alpha (RORα), germinal centre expressed transcript 2 (GCET2) and chitinase 3-like protein 2 (CHI3L2) on admission was associated with beneficial treatment response. In addition, leukocyte-specific protein 1 (LSP1) significantly decreased after 5 weeks of treatment in MDD responder patients.

Furthermore, in another interesting study, Duric et al.^117^ provided new evidence that disruption of synaptic and glutamatergic signalling pathways contributes to the pathophysiology of MDD by examining the genetic profile of micro-dissected subfields of postmortem hippocampus from MDD subjects. The authors found a significant dysregulation of synaptic function/structure related genes Synaptosome Associated Protein 25 (SNAP25), Disks large homolog 2 (DLG2), Microtubule-associated protein 1A (MAP1A) and 2-amino-3-(5-methyl-3-oxo-1,2-oxazol-4-yl) propanoic acid receptor subunit genes GLUR1 and GLUR3.

Finally, a recent study^118^ has performed genome-wide gene expression analyses in depressed patients prospectively divided in responders and non-responders to an 8-week trial of escitalopram treatment. The authors have found two genes exhibiting an increase in their mRNA expression levels in the non-responders group: CHN2 and JAK2. Specifically, CHN2 could alter the hippocampal neurogenesis, whereas JAK2 activates both innate and adaptive immunity, indicating that these genes could be possible candidate predictors of the treatment response.

The above studies not only have confirmed previous findings (such as an association between abnormalities in the immune and stress response as well as in neuroplasticity and neurotransmitters pathways and MDD), but they have also shown the huge advantage of performing whole-genome transcriptome assays to identify pathways and molecular mechanisms that are altered in MDD by using a hypothesis-free approach.

## Conclusions

In this review we have presented several studies which have investigated the expression levels of different genes in MDD patients, mostly obtained from whole blood but also from isolated mononuclear cells, isolated monocytes and postmortem brain tissues. Altogether these studies have identified a pattern of altered expression in several genes belonging to different biological systems such as inflammation, neurotransmission, HPA axis and neuroplasticity supporting data shown in our previous review published in 2013 by Hepgul et al.^15^.

In addition, to provide evidence supporting a personalized medicine approach to the treatment of MDD, we have reviewed studies that have analysed changes in gene expression levels associated with the treatment response. This association suggests that the gene expression approach, both hypothesis-driven and hypothesis-free, is particularly relevant from a clinical point of view as it allows to identify biomarkers that can help in the personalization of therapy and in the future development of novel intervention and treatment strategies.

Several studies have suggested that changes in gene expression measured in the blood mirror gene expression alterations occurring in the brain^11,119^ and a recent review of transcriptomic studies suggests that between 35% and 80% of known transcripts are present in both brain and blood tissue samples^119^. Moreover, Yan and colleagues have demonstrated the presence of an extracellular RNA at similar level both in the brain as well as in plasma samples^120^.

Another important aspect to consider is that peripheral blood biomarkers should be considered also in a different way. Indeed, in the clinical setting it is more important the clinical predictive value of a molecule instead of demonstrating the presence of its levels in a similar way both in the periphery and in the brain. In this context, inflammatory mediators, especially when measured in terms of mRNA levels, are a great example of how they can be used as predictors of treatment response in patients with MDD without being worried about the presence of similar levels in the brain. Hence, blood could serve diagnostic/prognostic purposes for MDD through profiling peripheral gene expression levels in blood cells^11,121^.

On the other hand, it is important to consider the difficulties in finding appropriate biomarkers, considering the heterogeneity of MDD. Indeed, some of the alterations associated with the disease might be influenced by several factors including childhood trauma, sex differences, lifestyle and demographic variables. For instance, it has been shown that trauma in childhood can lead to long-lasting effects on peripheral inflammation later in life, such as increased pro-inflammatory cytokine levels^122,123^. Moreover, it has been shown that, although depressive symptoms are associated with inflammation, this association is highly influenced by race and gender^124^. Therefore, large cohorts characterized by all these factors could allow the identification of peripheral biomarkers associated with specific endophenotypes of depression and associated with specific clinical variables known to influence also treatment response.

Altogether, these data have shown that the measurement of gene expression levels can be particularly helpful in the clinical setting, for an early prediction of treatment response in MDD patients. Indeed, as widely discussed in this review, mRNA biomarkers can predict the antidepressant response when measured at baseline or they can be useful in monitoring the efficacy of the treatment when measured during the therapy. This could lead to an improvement in the antidepressant response not only with a benefit for depressed patients, but also with a reduction of the associated health care costs.

However, to our knowledge, no gene expression biomarkers have been translated into the clinical practice yet, since most of the available studies have often used assays that are laboratory-specific and that are mainly based on a relative rather than absolute quantification.

Briefly, the relative quantification method compares the expression levels of a target gene in one group to those in an another group, for example patients to controls, using internal controls (housekeeping genes) for normalization. However, this relative gene expression based approach, although helpful in the identification and prioritization of novel biomarkers associated with antidepressant treatment, cannot be reflected as a routine into the clinical practice.

Conversely, an absolute gene expression quantification based approach could represent the best one to be implemented in the clinical setting, as it may help clinicians to predict and monitor the antidepressant response in a shorter time. Indeed, the absolute quantification is based on a standard curve, which is prepared from samples of known template concentration. Then, the concentration of any unknown sample can be determined by simple interpolation of its signal into this standard curve. Interestingly, because of these standard parameters, absolute mRNA values allow to establish given thresholds that are more likely to be individually measured and that are more comparable across different laboratories. For example, in a possible clinical setting scenario, the quantification of an absolute expression of certain biomarkers that, like cytokines, can predict the treatment response, can provide real-time information on the status of those biological factors that can influence treatment response. For example patients whose absolute mRNA values of pro-inflammatory cytokines are below the suggested cut-off could receive standard care treatment with conventional antidepressant drugs, whereas patients with absolute mRNA values higher than the suggested cut-off could be early directed toward more assertive antidepressant strategies, with patients receiving from the beginning a combination of antidepressant drugs, or adjuvant therapies such as anti-inflammatory drugs to pushdown the inflammatory status making the antidepressant therapies more efficacius.

Thus, an absolute quantification of gene expression biomarkers could avoid exposing depressed patients to unnecessary pharmacological strategies based on a try-and-error approach.

In conclusion, the absolute quantification of gene expression biomarkers represent the best approach to focus on in the next few years, to implement gene expression measurement in the clinical setting.
